# Effective Collection and Detection of Airborne Species Using SERS-Based Detection and Localized Electrodynamic Precipitation

**DOI:** 10.1002/adma.201300472

**Published:** 2013-05-31

**Authors:** En-Chiang Lin, Jun Fang, Se-Chul Park, Thomas Stauden, Joerg Pezoldt, Heiko O Jacobs

**Affiliations:** 1 University of Minnesota, Electrical and Computer EngineeringRm. 4-178, 200 Union St. SE, Minneapolis, MN 55455, USA; 2 Fachgebiet Nanotechnology, Ilmenau University of TechnologyGustav-Kirchhoff-Strasse 1, D-98693 Ilmenau, Germany

Detection of airborne species has attracted much attention due to the applications and potential for explosives detection and environmental monitoring.^1^–^4^ The detection of chemical agents commonly requires sensing schemes where the analytes are absorbed on a surface. The process of transport, absorption and precipitation is therefore critical to the detection limit of the analytes. This is true for all established gas phase sensing concepts, including gas chromatography,^5^ ion mobility spectrometry (IMS),^6^, ^7^ mass spectrometry,^8^ metal-semiconductor-metal-based sensors,^1^, ^9^, ^10^ chemical field-effect transistors,^11^ nanocantilever,^12^ infrared detection,^13^ and surface-enhanced Raman spectroscopy (SERS).^14^, ^15^

Recently Schedin et al. reported gas molecule detection with a sensitivity down to a single-molecule level^16^ and used diffusion as a mechanism for transport. While the results are impressive, the use of diffusion may not be the best approach, since it leads to a collection efficiency of the airborne species which is not optimized. In other words, single-molecular detection sensitivity is important, but requires the molecule of interest to reach the sensing surface with a size of possibly less than 100 nm. Effective collection on a small sensing area is not possible based on diffusion alone and the employment of a directed force will be required to solve the problem of transport. This transport problem could be addressed for example using thermophoretic and Coulomb forces to transport the analytes from a distance away to the sensing surface. At practical temperature gradients, the thermophoretic force, however, remains low compared to concepts that use electrostatic precipitation.^4^

Different from prior diffusion-based collection methods,^5^–^15^ this article reports and applies a localized electrodynamic precipitation concept to collect, spot, and detect airborne species at higher rates. Molecules are directed from a space that is centimeters away to specific sensing regions and areas with 100 nm control over the lateral position and spot size. The detection scheme is demonstrated using a surface-enhanced Raman spectroscopy (SERS) sensitized nanostructured surface including the standardized “Ag film over nanosphere (AgFON) substrate”.^14^, ^17^ In total, this study compares three different analyte delivery concepts (standard diffusion, global electrodynamic precipitation, and localized nanolens based precipitation) and three different SERS enhancement layers (a flat silver film, a nanolens enabled localized deposited film of silver nanoparticles, and the standard AgFON surface layer). The electrodynamic nanolens array reported here is a new design element. The nanolens array enables us to funnel and concentrate the airborne analyte molecules to discrete sensing points with sub-100 nm lateral resolution. The introduction of this concept had the biggest impact in terms of increasing the SERS signal intensity; a factor of 633 when compared to a standard mechanism of diffusion was observed. The nanolens array was also used to direct the precipitation of Ag nanoparticles to prepare a SERS enhancement layer which performed equally well as the (AgFON) standard.

**Figure** **1** details the delivery methods and test structures employed to evaluate and demonstrate advanced collection, spotting, and detection of airborne species, testing three different delivery mechanisms (a,b,c) and three different SERS nanostructured surface-layer designs (c,d,e). Domain 1 depicts the conventional concept of diffusion-based collection utilizing a conducting AgFON “SERS standard” sensor surface. This represents the state of the art and experimental approach that is commonly used. Airborne analyte molecules such as the depicted benzenethiols molecules attach to the surface over time on the basis of diffusion. However, aerosols always contain a fraction of charged species. While this fraction is commonly at least of factor of 1000 smaller than neutral particles it might be possible to manipulate these far fewer molecules more effectively, which is explored in this study through the application of an external potential to electrically separated domain electrodes, labeled as domains 2–5. Domains 3–5 also use the application of an external bias voltage, but add a localized electrodynamic collection approach, whereby a nanolens is formed using a patterned positively charged photoresist layer (PR) to funnel, concentrate, and spot charged molecules to predetermined locations. The general idea of a nanolens is to use a charged resist, which influences the trajectory of charged material. The resist is insulation and blocks charge dissipation. The opening to the biased domain electrode provides the only location where a charged material flux can be established under steady-state conditions. This approach is a novel design element since it achieves localized collection of charged analyte molecules at a higher level of concentration than otherwise possible. While domains 3–5 use the same nanolens directed collection approach the SERS sensor surfaces is adjusted to be different: (c) depicts the AgFON “SERS standard” composed of Ag coated 200 nm in diameter closed packed silica beads, (d) a SERS surface layer composed of localized aggregated (5–10 nm) Ag particles forming a 250 nm thick deposit, and (e) a SERS reference layer composed of a bare and flat 150 nm-thick silver surface, which provides another reference. The inserted SEM micrographs next to the schematics show the actual dimensions of the fabricated test structures.

**Figure 1:**
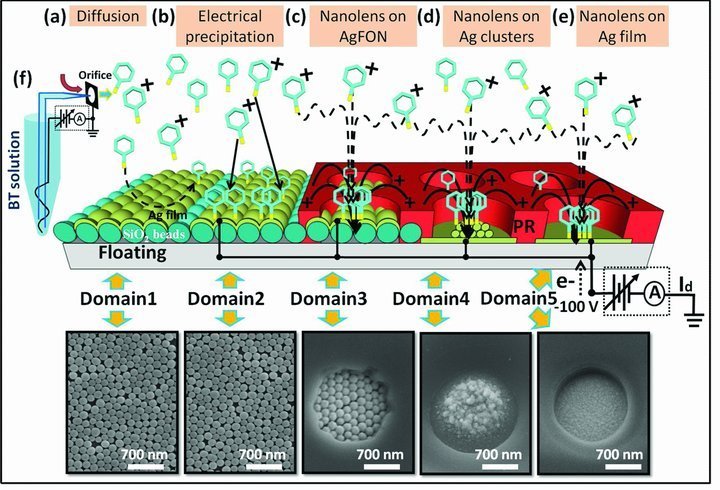
Methods and test samples employed to evaluate and demonstrate advanced collection, spotting, and detection of airborne species testing three different delivery mechanisms (a,b,c) and three different SERS nanostructured surface layer designs (c,d,e) on a single substrate in a single experiment. (a) Schematic depicting analyte precipitation on the Ag coated SERS surface on the basis of diffusion (domain 1). (b) A fraction of the anayte is charged and captured through application of a bias to the conducting AgFON domain (domain 2). (c) A nanolens array composed of 1 μm in diameter openings inside a 500 nm thick positively charged resist is used to funnel the charged analyte to predetermined locations leading to a locally enhanced capture rate and concentration (domain 3). Domains 1–3 depict a layer of Ag coated 200 nm in diameter closed packed silica beads referred to as AgFON standard. (d) The same nanolens based collection is maintained but the AgFON sensor surface is replaced with localized aggregated (5–10 nm) Ag particles forming a 250 nm think deposit on a biased flat silver layer (domain 4), and (e) a biased flat silver domain which provides a reference (domain 5). The SEM micrographs depict the actual structures that were used in the experiments. (f) Testing environment depicting electrospray ionization to produce a flux of neutral and charged analytes of known concentration.

To test this substrate and the collection concepts, we used a commercially available electrospray system (TSI Inc., Model 3480), which is capable of preparing various aerosols containing a known composition of charged and neutral analyte molecules (Figure 1f). In brief, the system consists of a high-voltage source, a pressure regulator, a pressure chamber, a capillary, and the electrospray chamber. The pressure chambers house a centrifuge vial, a high-voltage platinum electrode, and a fused silica capillary, which carries the solution out into the electrospray chamber using 1.25 atm pressure. The following conditions were used. A positive electrospray voltage is increased until the extruded liquid (50 nL/min) forms a cone shape, known as cone-jet mode,^18^ which leads to rapid evaporation in close proximity to the orifice and an aerosol containing charged molecules (used light blue for benzenethiol in Figure 1). In positive ion mode the process produces in addition to neutral molecules, positively charged molecules.^19^ Of importance in our experiment are benzenethiol ions (C_6_H_5_SH_2_^+^), solvent ions (C_2_H_5_OH_2_^+^), and nitrogen ions (N_2_^+^).

The guided nanolens assisted deposition process depicted in (Figure 1c–e) should be considered as an electrodynamic process since the field distribution evolves over time. In the initial stages of the experiment, ions respond to the external bias voltage. The smallest ions, N_2_^+^, have the highest mobility and arrive first at the sample surface. This transient response results in a sheath of space charge on the sample surface, depicted as “+” on the red-colored photoresist layer, which alters the potential distribution. The potential distribution equilibrates and leads to a potential funnel where the analyte deposits in the center of the opening. Steady-state charge dissipation can only occur in the opening and leads to a measurable flux of positive gas ions, which includes the targeted molecules under a negative substrate bias of −100 V, and this flux can directly be recorded using electrometers (Keithley 6517A) marked with the letter “A”.

In the experiments, we used a benzenethiol solution (liquid density = 1.073 g/mL, molecular weight = 110 g/mol) which was diluted in a 1:1 volume ratio with ethanol; secondly, the solution is sprayed at a rate of 50 nL/min, which translates to 2.43 × 10^−7^ *N*_A_ benzenethiol molecules per minute where *N*_A_ is Avogadro's constant; third it is mixed with 1 L/min nitrogen carrier gas at 1.25 atm adding 4.1 × 10^−2^ *N*_A_ nitrogen molecules per minute to the mixture; at this stage the analyte is diluted down to 5.9 ppm. Charging occurs as a result of the electrospray process, and the amount of charge can calculated directly from the electrospray current. All of the experiments were conducted using a typical electrospray current of 100 nA, which represents an ion-current flux of 6.23 × 10^−11^ *N*_A_ elementary charges per minute. In relative terms, these numbers translate to a gas mixture which is composed of approximately 1.5 ppb charged molecules (calculated using 6.23 × 10^−11^ *N*_A_ charged molecules in 4.1 × 10^−2^ *N*_A_ nitrogen molecules) and 5.9 ppm neutral benzenethiol molecules (calculated using 2.43 × 10^−7^ *N*_A_ benzenethiol molecules in 4.1 × 10^−2^ *N*_A_ nitrogen molecules). The given values are upper limits, since downstream mixture and charge-exchange reactions before the mixture reaches the substrate, which is placed 5 cm away from the nozzle, are not included. From a relative comparison there are approximately 3 orders of magnitudes less charged benzenethiol molecules (<1.5 ppb) in the gas mixture than neutral ones (<5.9 ppm).

A goal was to prepare a single substrate with different domains and reference structures to aid in the comparative study of the design elements and to help get conclusive results. **Figure** **2** depicts fabrication details of the investigated designs. The preparation followed these steps: glass slides were pretreated in Piranha etches at 120 °C for 30 min and treated in a 5:1:1 ratio of H_2_O:NH_4_OH:H_2_O_2_ for 30 min to make the surface hydrophilic. Monodisperse silica nanospheres (200 nm in diameter) were assembled into a closely packed layer onto a portion of the substrate through drop coating using 4% silica spheres by weight in water further diluted in ethanol (1:1 volume ratio) to aid in the spreading (Figure 2a). We used 20 nm and 150 nm e-beam-coated chromium and silver, respectively, and hand-placed capillaries 100 μm in diameter to fabricate 5 electrically isolated domains on a single substrate (Figure 2b,c). The depicted metal coated silica spheres are known in the literature as the “AgFON SERS standard”.^14^, ^17^ The silver film provides a conductive layer. This conductive layer allows for the application of an external bias voltage, which is used to evaluate if a field-driven approach can increase the collection efficiency of charge molecules, when compared to prior concepts where the rate of absorption was driven by diffusion and where the substrate was left floating.^14^, ^15^ Domains 3–5 were further modified through integration of a photoresist based nanolens array (Figure 2d) using a 500 nm-thick spin coated photoresist layer (Microposit S1805), which was patterned using photolithography to define a 1 μm in diameter and 3 μm pitched hole pattern. The SEM image depicts the actual test structure and dimensions that were used.

**Figure 2:**
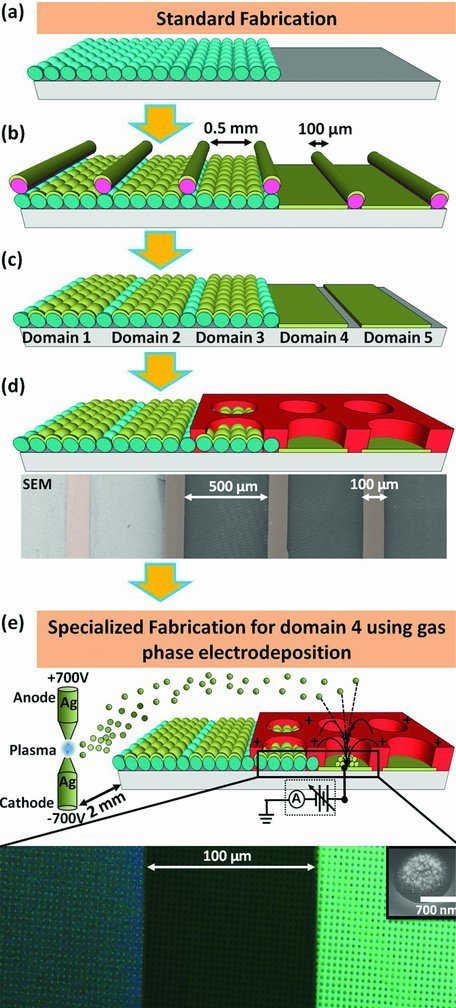
Schematics and micrographs detailing the fabrication of a programmable SERS substrate for advanced collection, spotting and detection of airborne species. (a) 200 nm silica nanospheres are locally assembled on a glass slide to form a closely packed layer of spheres. (b and c) Five electrically separated domains are formed using a vapor coated metal layer (20 nm Cr and 150 nm Ag); hand placed 100 μm in diameter capillaries mask the areas between individual domains. (d) A spin coated and patterned photoresist layer is used to define 1 μm in diameter circular openings to the underlying electrodes; the layer is limited to domain 3–5. (e) Gas phase electrodeposition^20^–^22^ is used to selectively deposit localized aggregated (5–10 nm) Ag particles in the openings on domain 4 (see text for details). Inserts in (d,e) depict SEM and optical microscope images of the corresponding test structures and dimensions that were used.

The last step applies a thin layer of silver nanoparticles to domain 4 (Figure 2e). This particular process has only been reported recently in the literature.^20^–^22^ In brief, the process is referred to as gas-phase electrodeposition, since it uses charged nanoparticles in a carrier gas that can be applied locally through application of an external bias voltage.^20^–^22^ It resembles electrodeposition in the liquid phase, but replaces the liquid medium with a carrier gas. Here, it is used to deposit (5–10 nm) Ag particles locally on domain 4, through application of a negative bias voltage of 150 V, yielding a 250 nm-thick aggregated layer of Ag nanoparticles in 60 s at 100 °C on only the biased domain. The origin of the charged particles is a confined “DC-arc-discharge-based plasma” which uses two consumable electrodes. Experimentally we used the following conditions: 0.5 mm-spaced silver electrodes, a discharge current of 100 mA in air at 1 atm, and a distance of 2 mm to the nearby substrate. The actual appearance of the localized nanoparticles is shown in the SEM (insert) whereby the optical microscope image provides an overview of the corresponding test structures with the relevant dimensions.

**Figure** **3** compares the recorded SERS signals testing a 20 s-long exposure to the gas mixture containing charged (<1.5 ppb) and neutral benzenethiol (<5.9 ppm) molecules in nitrogen. The actual biasing conditions are depicted in Figure 3a. The resulting intensity map (Figure 3b) of the Raman peak at 1075 cm^−1^ and the SERS spectra (Figure 3c) were recorded using identical recording conditions. In order to get the average SERS spectrum instead of individual hotspot spectrum, the average was recorded over identical windows outlined using the white dashed lines in Figure 3b. The spectrum for the unbiased case shows a weak signal and the detection of the uncharged benzenethiol was difficult at 6 ppm; the characteristic peak at 1075 cm^−1^ is recorded with 0.3 counts per unit area, which means very few benzenethiol molecules are collected on this substrate. The signal increases to 103 counts per unit area for the biased AgFON substrate and to 190 counts per unit area for the biased AgFON substrate with integrated nanolens array, which represents a factor of 633, comparing the biased nanolens array with the unbiased AgFON standard. The result is particular intriguing if one considers that we had at least 3 orders of magnitudes fewer charged benzenethiols (<1.5 ppb) in the gas mixture than neutral ones (<5.9 ppm). Or in other words, trace amounts of molecules at a concentration of 1.5 ppb can be detected in 20 s as long as they are charged, which is not possible using a standard mechanism of diffusion where a concentration of 6 ppm is required to get above the noise level of the instrument. The intensity increase by a factor of 633, in combination with the fact that less than 1 out 3900 analyte molecules is likely to be going to be charged, can further be used to provide a rough estimate for the capture efficiencies; specifically, the two numbers suggest that the capture efficiency of charged molecules may be 6 orders (considering 633 × 3900) of magnitudes higher than neutral ones in the current design.

**Figure 3:**
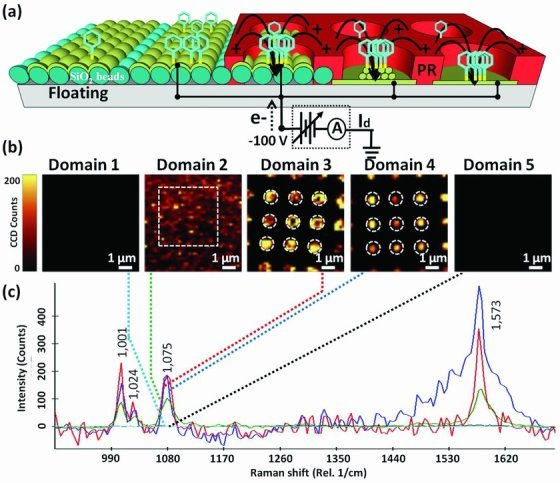
Schematics (a), with corresponding scanning confocal Raman microscopy intensity map (b), and spectra (c), comparing different delivery methods and SERS sensor designs on a single substrate in a single experiment which is exposed to a gas mixture containing both charged (<1.5 ppb) and neutral benzenethiols (<5.9 ppm) for 20s. The results represent standard diffusion (Domain 1), a biased region (Domain 2) as well as a nanolens enabled molecular spotting approach integrated on top of a AgFON SERS standard layer (Domain 3), a localized Ag nanoparticle layer (Domain 4), and a flat silver layer (Domain 5).

Domains 3–5 in Figure 3 compare the three different SERS sensor designs. Domain 4 introduces a SERS layer which is composed of localized aggregated Ag nanoparticles. Considering this layer, we found no noticeable difference considering the peak intensity at 1075 cm^−1^, yielding 191 counts, which is almost identical to the value recorded using the nanolens-enhanced delivery on the AgFON SERS surface layer (Domain 3). Domain 5 was used as a reference, and was composed of a flat silver film underneath the nanolens array. The flat silver reference electrode showed almost no (0.2 counts) detectable peaks confirming the absence of surface enhanced plasmonic resonances in this film. Considering this set of results, we conclude that the loosely aggregated Ag nanoparticle (Domain 4) is equally well suited as an enhancement layer as the AgFON standard (Domain 3). The advantages of the loosely aggregated Ag nanoparticle (Domain 4) are that it can be deposited into desired locations and that it may enable multiplexing of various materials in further studies. We repeated this experiment five times. The nanolens-based collection region (Domain 3 and 4) produced the highest counts in each case; the intensity factor varied by 6% (STD) between experiments.

Overall the nanolens approach has the advantage that it supports a more automated analysis, since it eliminates the searching and hand-picking of hotspots, which is a common practice in SERS-related measurements. Instead, it uses a standard array of detection windows outlined with the dashed lines to overcome the problems associated with the handpicking of hotspots.

In conclusion, various nanostructured sensors currently aim to claim single-molecular detection by a reduction of the active sensor size. An equally important challenge, however, can be found in the question as to “whether the analyte will find the nanometer-sized surface”. The reduction in the size of the active sensor will ultimately require research on methods that enable localized delivery. The reported electrodynamic collection concept is a first step in this direction. The process can be applied to small molecules of almost any type, as long as they can be charged. The use of electrodynamic forces benefits from unique scaling laws which can be both long range using parallel plates, and highly localized with sub-100 nm confinement using simple patterned insulating resists that can be charged to guide the analyte to specific locations. This is not possible with any other method. While the collection and spotting is demonstrated using surface-enhanced Raman spectroscopy, the localized gas-phase analyte delivery method should be applicable to other sensing concepts. The relative sensitivity increases in the case of the SERS sensor was 633 comparing the biased nanolens array with the unbiased AgFON standard. This value has not be optimized and is based on the 1 μm diameter and 3 μm pitch hole pattern; higher values can be anticipated but would require optimization of the opening size, pitch, domain size, and domain potential. Most interesting, however, is the fact that the gas mixture contained far fewer charged (<1.5 ppb) analyte molecules than neutral ones (<5.9 ppm); in other words the inability to detect the neutral molecules despite the fact the concentration was 4 orders of magnitude larger than the charge molecules motivates the anticipated gains of incorporating advanced charging concepts from a sensor system point of view. Many gas-sensor systems currently use diffusion as the only mechanism of transport, and dramatic improvements can be anticipated incorporating various forms of localized delivery, which would impact environmental monitoring systems or the detection of chemical or biological warfare agents. One of the reported SERS domains was functionalized using Ag nanoparticles, which assembled at precise locations in the photoresist openings, on the basis of programmable localized precipitation. This type of localized functionalization provides an alternative to the AgFON standard. It also illustrates that the process is not only applicable to molecules. Considering the present results further it is possible to envisage a multiplexed sensing platform where analytes or nanoparticles of different types are localized in an active matrix like fashion.

## Experimental Section

*Materials*: All chemicals were reagent grade and used as received. Surfactant-free, silica nanosphere suspensions (200 nm, 4 wt%) were acquired from Bangs Laboratories, Inc. Benzenethiol (BT) was purchased from Sigma–Aldrich (Milwaukee, WI). NH_4_OH, H_2_O_2_, and H_2_SO_4_ were purchased from Fisher Scientific (Fairlawn, VA).

*SERS Spectra*: The SERS spectra and 2D confocal-microscopy intensity maps were acquired using a near-IR confocal Raman microscopy system (Witec Alpha 300R). A fiber-optic-interfaced 514 nm diode laser was used as the laser source. The laser power was maintained constant and set to ≍2 mW within one experimental set of measurements. The raster and spot size considering the optics is approximately 300 nm. The collection time for each raster spot constant was 1 s. SERS spectra were collected from multiple spots across the substrate and from multiple substrates.

*Characterization*: A scanning electron microscope (JEOL 6500) was used to examine the surface morphology of the various domains. Transmission electron microscopy (FEI Tecnai F30) was used to determine the size and morphology of the Ag nanoparticles which deposited on domain 4 through gas phase electrodeposition. The reflectance absorption spectrum was analyzed using an optical fiber vis–NIR spectrophotometer (Ocean Optics, USB4000 VIS-NIR spectrometer, QR400-7-UV–vis reflection probe). The reflectance absorption spectrum of AgFON was collected and used for the chosen wavelength (514.5 nm).

## Supplementary material

As a service to our authors and readers, this journal provides supporting information supplied by the authors. Such materials are peer reviewed and may be re-organized for online delivery, but are not copy-edited or typeset. Technical support issues arising from supporting information (other than missing files) should be addressed to the authors.
