# Supplementary material for: Effective Collection and Detection of Airborne Species Using SERS-Based Detection and Localized Electrodynamic Precipitation
Source: Adv Mater. 2013 May 31;25(26):3554–9. doi: 10.1002/adma.201300472 (PMC3759703; doi:10.1002/adma.201300472)
Supplement: Supplementary file 1 [file adma0025-3554-SD1.pdf]

Copyright WILEY-VCH Verlag GmbH & Co. KGaA, 69469 Weinheim, Germany, 2013.

ADVANCED MATERIALS

Supporting Information

for *Adv. Mater.*, DOI: 10.1002/adma.201300472

Effective Collection and Detection of Airborne Species Using SERS-Based Detection and Localized Electrodynamic Precipitation

*En-Chiang Lin, Jun Fang, Se-Chul Park, Thomas Stauden, Joerg Pezoldt, and Heiko O. Jacobs**

Supporting Information

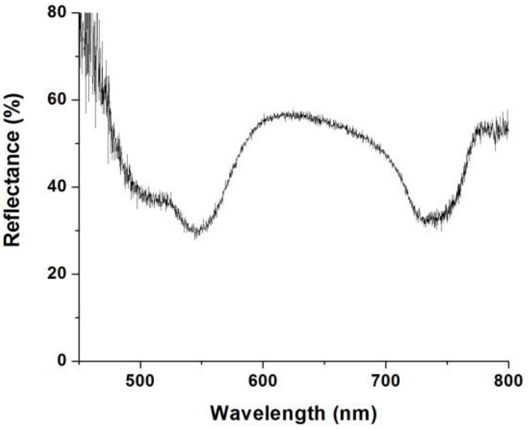

Fig. S1 The reflectance absorption spectrum of AgFON was collected and used for the chosen wavelength (514.5 nm).
